# Genetic tools for the investigation of *Roseobacter *clade bacteria

**DOI:** 10.1186/1471-2180-9-265

**Published:** 2009-12-18

**Authors:** Tanja Piekarski, Ina Buchholz, Thomas Drepper, Max Schobert, Irene Wagner-Doebler, Petra Tielen, Dieter Jahn

**Affiliations:** 1 Institute of Microbiology, Universität Braunschweig, Spielmannstrasse 7, D-38106 Braunschweig, Germany; 2 Department for Cell Biology, Research Group Microbial Communication, Helmholtz Center for Infection Research, Inhoffenstrasse 7, D-38124 Braunschweig, Germany; 3 Institute of Molecular Enzyme Technology, Heinrich-Heine- University Düsseldorf, Research Center Jülich, Stetternicher Forst, D-52426 Jülich, Germany

## Abstract

**Background:**

The *Roseobacter *clade represents one of the most abundant, metabolically versatile and ecologically important bacterial groups found in marine habitats. A detailed molecular investigation of the regulatory and metabolic networks of these organisms is currently limited for many strains by missing suitable genetic tools.

**Results:**

Conjugation and electroporation methods for the efficient and stable genetic transformation of selected *Roseobacter *clade bacteria including *Dinoroseobacter shibae*, *Oceanibulbus indolifex*, *Phaeobacter gallaeciensis*, *Phaeobacter inhibens*, *Roseobacter denitrificans *and *Roseobacter litoralis *were tested. For this purpose an antibiotic resistance screening was performed and suitable genetic markers were selected. Based on these transformation protocols stably maintained plasmids were identified. A plasmid encoded oxygen-independent fluorescent system was established using the flavin mononucleotide-based fluorescent protein FbFP. Finally, a chromosomal gene knockout strategy was successfully employed for the inactivation of the anaerobic metabolism regulatory gene *dnr *from *D. shibae *DFL12^T^.

**Conclusion:**

A genetic toolbox for members of the *Roseobacter *clade was established. This provides a solid methodical basis for the detailed elucidation of gene regulatory and metabolic networks underlying the ecological success of this group of marine bacteria.

## Background

The *Roseobacter *clade is a lineage of the *Rhodobacteraceae *within the *Alphaproteobacteria*. It is the most abundant and diverse group of marine Gram-negative, non-obligately phototrophic prokaryotes. They represent up to 25% of marine communities, especially in coastal and polar regions [reviewed in [1,2]]. Currently, 41 subclusters are described, covering all major oceanic habitats like seawater, algal blooms, microbial mats, sediments, sea ice and marine invertebrates [2]. Members of the *Roseobacter *clade display diverse physiologies. For example, some members can generate energy via aerobic anoxygenic photosynthesis, oxidize the green-house gas carbon monoxide and produce the climate-relevant gas dimethylsulfide through the degradation of different sulphur compounds. Thereby, these bacteria significantly influence the global carbon and sulphur cycles as well as the climate [2]. Moreover, they are able to degrade aromatic compounds, reduce trace metals, produce bioactive secondary metabolites, perform quorum sensing and can establish symbiotic and pathogenic relationships [1-5]. Several members of the *Roseobacter *clade have been implicated as causative agents of juvenile oyster disease in Eastern oyster and black band disease in scleractina coral [2,6], or were described as probiotics for fish larvae [7,8].

Scientific interest in this bacterial group increased steadily since the description of its first representatives *Roseobacter denitrificans *and *Roseobacter litoralis *[9]. Since the first genomes of *Silicibacter pomeroyi *and *R. denitrificans *have been completely elucidated [10,11] a massive genome sequencing approach financed by the Gordon & Betty Moore foundation resulted in currently 23 draft and 5 finished genome sequences from the *Roseobacter *clade. Some of these sequences are organized in the *ROseobacter *SYstems biology database (ROSY) which allows the comparison of the genome data and supplies an integrated platform for comparative genomics and systems biology approaches [http://rosy.tu-bs.de/; [12]].

Many *Roseobacter *strains, including *R. denitrificans*, *R. litoralis*, *Dinoroseobacter shibae *and *S. pomeroyi *carry plasmids of different size [13,14]. They range from 4.3 kb to 821.7 kb and can carry up to 20% of the genome content [4]. Therefore, due to possible incompatibilities, the choice of suitable vectors for genetic investigations is of enormous importance [15].

The availability of the complete genome sequences of this important group of bacteria is a crucial prerequisite for a detailed analysis of their physiological and ecological properties. However, for systems biology approaches suitable methods allowing easy and efficient genetic manipulation of these strains are needed. Such techniques are already established for other members of the *Rhodobacteraceae*, including *Rhodobacter sphaeroides *and *Rhodobacter capsulatus *[e.g. [16-18]]. However, in this context only little is known for members of the *Roseobacter *clade. Techniques for electroporation, transposon mutagenesis, biparental mating, gene knockout and genetic complementation were described only for *Silicibacter *sp. TM1040 [19,20], *S. pomeroyi *[21,22] and *Sulfitobacter *sp. J441 [23]. In the latter study, also *lacZ *reporter gene fusions were constructed for gene expression analyses. Moreover, transposon mutagenesis of *Phaeobacter sp*. was described [19]. However, already in 2005, the *Roseobacter *clade comprised a large phylogenetic diversity with 36 described species representing 17 genera [6]. In the meantime, many more species have been described, making it increasingly difficult to obtain stable tree topologies based on 16S rRNA sequences [4]. It is well known from other bacterial groups that genetic tools developed for one genus do not work in a related genus or even in a different strain of the same species. Therefore, we systematically determined key parameters required for successful genetic experiments in strains which cover phylogenetic groups complementary to the few already studied. We selected *R. litoralis *and *R. denitrificans*, the archetypical isolates from the *Roseobacter *clade whose physiologies have been studied for a long time. Moreover, *Oceanibulbus indolifex*, a non phototroph which is related to *Sulfitobacter *was selected. All three species are in the middle of the *Roseobacter *radiation [4]. Furthermore, we selected two species of *Phaeobacter *(formerly *Ruegeria*). Finally, *D. shibae *a genus which is at the base of the *Roseobacter *radiation, was studied in more detail.

We first investigated the antibiotic susceptibility of the selected *Roseobacter *clade species to identify useful selective markers. Using these antibiotic markers, we tested transformation and conjugation methods using plasmid-DNA transfer with different classes of plasmids. A reporter gene fusion system was established based on a conjugative and stably maintained plasmid encoding a flavin mononucleotide-based fluorescent protein gene. Finally, a gene knockout strategy was successfully applied in *D. shibae*.

## Results and Discussion

### Differential growth of *Escherichia coli *and *Roseobacter *strains in response to varying salt concentrations in the culture medium

Aim of this study was to test genetic methods, applicable for the investigation of selected representative *Roseobacter *clade bacteria. Tools of interest include a gene knockout system, a plasmid-based system for homologous gene expression and complementation of gene defects *in trans*, and a reporter gene system. So far, such genetic methods were described for only a few members of the *Roseobacter *clade as *Silicibacter *and *Sulfitobacter *[19-23]. Certainly it is unknown if these genetic methods are also applicable for other representative members of the huge *Roseobacter *clade. Therefore, we tested these and other methods on several members of the *Roseobacter *strains spread over the whole radiation of this clade and thereby formed a very physiologic diverse subgroup. In the context of genetic methods, the selection of antibiotic resistance markers is the basis for bacterial genetics and molecular biology. However, marine bacteria of the *Roseobacter *clade require appropriate salt concentrations for sufficient growth. Since several antibiotics are inactive at high salt concentrations, first a suitable growth medium for resistance screening had to be identified. Generally, the standard growth medium for bacteria of the *Roseobacter *clade is Marine Broth (MB) [4,22,24]. However, MB restricts the survival of *E. coli*, which is used for plasmid-DNA transfer by biparental mating (see below). Therefore, we initially compared the growth of six marine bacteria (i.e. *P. gallaeciensis, P. inhibens, R. denitrificans, R. litoralis, O. indolifex, D. shibae*) and *E. coli *using five media with different salt concentrations (Table 1). As expected, bacteria of the *Roseobacter *clade have an absolute requirement for salts, including high concentrations of NaCl [4,25] and therefore did not grow in Luria Bertani (LB) medium. However, slow growth in LB-medium supplemented with 8.5 g sea salts (LB+hs) compared to MB was observed. On the other hand, the *E. coli *donor strain ST18 [26] grew in LB and even in LB+hs, but did not grow in high salt-containing media as MB and LB supplemented with 17 g sea salts (LB+s). Thus, only half-concentrated MB (hMB) allowed growth of all tested bacteria, albeit with partly decreased growth rates compared to their commonly used growth media.

**Table 1:** Growth rates of used strains in different media^a^

Strain	growth rate μ[h^-1^]				
medium	MB	hMB	LB	LB+s	LB+hs
*P. inhibens*	0.80	0.48	n.d.	0.50	0.37
*P. gallaeciensis*	0.70	0.62	0.01	0.37	0.50
*O. indolifex*	0.43	0.50	n.d.	0.26	0.29
*R. litoralis*	0.20	0.28	n.d.	0.27	0.13
*R. denitrificans*	0.60	0.30	0.02	0.22	0.19
*D. shibae*	0.14	0.32	n.d.	0.09	0.31
*E. coli *ST18	0.08	0.70	1.01	0.09	1.04

n.d. not detectable

^a^The *Roseobacter *strains were cultivated at 30°C and 200 rpm. *E. coli *was cultivated at 37°C and 200 rpm. For growth of *E. coli *ST18 the media were supplemented with 50 μg/ml ALA. The results represent the mean value of two independent experiments performed in duplicate. A standard deviation of up to 16% was observed.

It was reported that several antibiotics, including tetracycline and gentamicin, can be affected in their chemistry by high salt concentrations as found in MB [27]. For example, the aminoglycoside kanamycin chelates Cu^2+ ^[28] and tetracycline forms complexes with divalent cations such as Mg^2+^, Fe^2+ ^and Ca^2+^. These interactions have no significant impact on the stability of tetracycline, but decrease the membrane permeability of a cell and therefore the bioavailability of this antibiotic [27,29-31]. Up to ten times higher concentrations of gentamicin, carbenicillin, chloramphenicol and tetracycline were required for *Roseobacter *growth inhibition in MB medium (data not shown) compared to hMB with lower sea salt concentrations (see below). Control experiments with *E. coli *showed that all used antibiotics were active over the whole incubation time in hMB at chosen conditions (data not shown). Consequently, hMB medium was used for further investigations.

### Screening of *Roseobacter *clade bacteria for antibiotic susceptibility

The six different species of the *Roseobacter *clade were examined for their antibiotic susceptibility. Furthermore, seven strains of *D. shibae*, isolated from different marine sources, were tested for the degree of susceptibility difference within one species. Such strain-specific differences were already described for other species as *E. coli *[32], *Pseudomonas aeruginosa *[33] and other pathogens [34]. Table 2 represents the MIC in hMB medium after 72 h at 30°C. We tested antibiotics from different chemical groups, which are commonly used in molecular biology, such as tetracycline, chloramphenicol, the aminoglycosides kanamycin, gentamicin, streptomycin and spectinomycin as well as the two β-lactam antibiotics ampicillin and carbenicillin. Concentrations of up to 500 μg/ml were used. Bacteria able to grow above a concentration of 100 μg/ml of the respective antibiotic were defined as resistant.

**Table 2:** Susceptibility to antibiotics (Minimal inhibitory concentrations; MIC) of strains from the *Roseobacter *clade.

Strain/Antibiotic	Amp [μg/ml]	Carb [μg/ml]	Cm [μg/ml]	Gm [μg/ml]	Kan [μg/ml]	Spec [μg/ml]	Strep [μg/ml]	Tc [μg/ml]
*Phaeobacter inhibens *T5^T^	90	20	15	5	80	5	20	10
*Phaeobacter gallaeciensis *2.10	>100	40	10	30	50	5	20	30
*Oceanibulbus indolifex *HEL-45^T^	30	20	10	10	20	10	10	30
*Roseobacter litoralis *6996^T^	>100	<2	10	20	40	10	5	35
*Roseobacter denitrificans *7001^T^	>100	<2	30	150	>100	>100	160	50
*Dinoroseobacter shibae *DFL-12^T^	>100	>100	15	20	>100	20	25	40
*Dinoroseobacter shibae *DFL-16	>100	>100	15	35	100	10	30	30
*Dinoroseobacter shibae *DFL-27	>100	>100	10	20	>100	15	40	20
*Dinoroseobacter shibae *DFL-30	>100	>100	10	20	>100	15	30	30
*Dinoroseobacter shibae *DFL-31	>100	>100	15	20	>100	10	35	35
*Dinoroseobacter shibae *DFL-36	>100	>100	10	20	>100	15	25	30
*Dinoroseobacter shibae *DFL-38	>100	>100	15	20	>100	10	35	25

^a^The determination of the MIC was performed in hMB after 72 h at 30°C. Fresh antibiotic stock solutions (10 mg/ml) were made for every experiment. Test tubes were inoculated to an OD_578 _of 0.05 with over night cultures of the *Roseobacter *strains in MB at 30°C. The results represent the mean of three independent experiments performed in duplicate. Amp, ampicillin; Carb, carbenicillin; Cm, chloramphenicol; Gm, gentamicin; Kan, kanamycin; Spec, spectinomycin; Strep, streptomycin; Tc, tetracycline

All tested species showed different susceptibilities to the antibiotics (Table 2). As expected, the seven *D. shibae *strains followed the same trend, with slight variations. They were all resistant to the β-lactam antibiotics ampicillin and carbenicillin. The level of tolerance to ampicillin was up to 500 μg/ml. The *Phaeobacter *strains, *R. denitrificans *and *R. litoralis *also showed resistance to ampicillin, whereas, in contrast to *D. shibae*, they were sensitive to carbenicillin. Initially, we hypothesised, that the unexpected high ampicillin tolerance might occur due to instability of this antibiotic. It has been reported that ampicillin lost 28% of activity after 24 h at room temperature [30]. However, control experiments with the *E. coli *strain DH5α revealed complete activity of ampicillin even after incubation for five days at 30°C (data not shown).

Analysing the annotated genomes of the strains by BLAST search and functional predictions (for details see Methods section), we identified genes encoding for β-lactamases, indicating that they are widespread over the *Roseobacter *clade. They were also found in *R. denitrificans*, *R. litoralis*, *P. gallaeciensis*, *O. indolifex *and *D. shibae*. For the latter strain, three β-lactamases encoding genes were identified [using ROSY; [12]]. Thus, the inactivation of the antibiotics via degradation by β-lactamases seems to be an intrinsic resistance mechanism.

Susceptibility of the *Roseobacter *strains differed towards the four tested aminoglycosides. *R. denitrificans *showed no susceptibility to all tested aminoglycosides. In contrast *R. litoralis *and *P. gallaeciensis *were sensitive to this group of antibiotics. Growth of *P. inhibens *was inhibited by high concentrations of kanamycin, but the bacterium reacted very sensitive to spectinomycin and gentamicin. The *D. shibae *strains were resistant to kanamycin, but relatively sensitive to the three other aminoglycosides. *O. indolifex *was susceptible to all aminoglycosides.

The resistance to the aminoglycoside gentamicin was already reported by Shiba [1991] as one of the characteristic properties of *R. denitrificans*. The corresponding genome exhibits a gene encoding for a putative aminoglycoside phosphotransferase, a type of enzyme inactivating aminoglycosides via modification [using IMG; [35], and ROSY; [12]]. Putative aminoglycoside phosphotransferase genes were also found in the *D. shibae *genome and those of other *Roseobacter *clade species. Most of these strains contain two putative genes, except for *D. shibae *DFL12^T ^which exhibits 10 genes [using IMG; [35]]. This observation might explain the high kanamycin tolerance of *D. shibae*.

The MICs for tetracycline and chloramphenicol were in a range of 10 - 50 μg/ml and 10 - 30 μg/ml, respectively. None of the tested species showed resistance to these two antibiotics. In summary, we identified at least three antibiotics for every strain which are suitable as selective makers for use in molecular biology and genetic protocols. In the following experiments we used twice the amount of the MIC for the selection of plasmid-containing strains and for the maintenance of the plasmids within the *Roseobacter *strains. Several groups reported that the MICs of bacteria grown in liquid cultures can be lower than for the same bacteria grown on agar plates as biofilms [36,37]. Control experiments demonstrated that only plasmid-containing cells survived twice of the MIC via expression of the plasmid-encoded resistance gene. Also in case of differences between MICs determined in static liquid culture and in aerated liquid cultures, the use of twice of the MIC ensured selection of plasmid-containing cells.

### *Roseobacter *clade bacteria are resistant to common chemical transformation approaches

First, chemical transformation methods [38] were tested for the transformation of the various *Roseobacter *strains. Chemo-competent cells were prepared with CaCl_2 _and furthermore with RbCl_2_. Plasmid-DNA transfer experiments were carried out by mixing bacteria with 50 ng plasmid-DNA (pBBR1MCS), followed by a 30 min-incubation on ice and a subsequent 2 min heat shock at 42°C similar to the standard procedure for *E. coli *[38]. Transformation of *Roseobacter *strains led to no transformants, either with CaCl_2_-competent or with RbCl_2_-competent cells. No transformants were observed for any of the 12 tested strains. Similar observations were made for *Rhodobacter *strains, which are close relatives of the *Roseobacter *strains [39]. Only one successful approach was described for *R. sphaeroides *in 1982 [16]. Initial experiments using the published method did not lead to transformants of *Roseobacter *clade bacteria.

### Transformation of *Roseobacter *clade bacteria via electroporation

Since common chemical transformation methods as described by Sambroock et al. [1989] did not lead to successful DNA transfer in *Roseobacter *clade bacteria (see above), the electroporation method was tested. Electroporation was performed following the protocol of Miller and Belas [2006]. This method was successfully used for other members of the *Roseobacter *clade as *Silicibacter *sp. [19,20] and *S. pomeroyi *[22]. Salt-free cell suspensions were prepared by washing with 10% (v/v) glycerol in ultra-pure water. We tested the washing procedure with increasing numbers of separate washing steps. A minimal of 5 washing steps was necessary to effectively remove the salts from culture media, verified by a resulting optimal pulse length of 4.9 - 5.0 ms. The competent cells were subsequently frozen in liquid nitrogen and stored at -80°C. Under these conditions cells can be stored for about 3 weeks, except of *R. denitrificans*, which was viable only for a maximum of 1 week. We used 25 ng and 50 ng plasmid-DNA (pBBR1MCS), both resulting in similar transformation rates. Different pulse intensities were tested (1.5 - 3.0 kV). An intensity of 2.5 kV revealed the best results and was used for further experiments. The electroporation method was successful for all tested strains, although transformation rates differed between them. A maximum of 1 × 10^3 ^cfu/μg plasmid-DNA were observed for *P. inhibens *and *R. litoralis*. Slightly higher efficiencies of 1 × 10^4 ^cfu/μg plasmid-DNA were observed for *D. shibae *and *R. denitrificans*. Good efficiencies were observed for *P. gallaeciensis *with 1 × 10^5 ^cfu/μg plasmid- DNA and *O. indolifex *with an efficiency of 1 × 10^7 ^cfu/μg plasmid-DNA. Recently, an optimized electroporation method was described for the Gram-negative *P. aeruginosa *resulting in transformation efficiencies ranging from 10^7 ^to 10^11 ^cfu/μg plasmid-DNA [40]. These results are comparable with the efficiencies obtained in *O. indolifex*, indicating that our protocol is sufficient for the members of the *Roseobacter *clade. Although the transformation efficiencies are much less for most of the tested *Roseobacter *strains, this technique can be used as a fast and easy method to transfer plasmids into *Roseobacter *cells.

### Efficient conjugal transformation of *Roseobacter *clade bacteria

Biparental mating using *E. coli *S17-1 as donor strain was described for plasmid transfer into *S. pomeroyi *and *Sulfitobacter *before [21,23]. Thereby, the use of spontaneous emerged antibiotic-resistant mutants of the recipient strains is one of the principles used to counter-select against the *E. coli *donor strain after conjugation [e.g. [23,41]]. It is well known that such mutations may also cause indirect pleiotropic effects that might influence the general physiology of the target strain. Changes in growth behaviour, uracil sensitivity and bacteriophage sensitivities were reported for spontaneous rifampicin-resistant mutants [42,43]. A second approach utilises auxotrophic donor strains. Here, we used *E. coli *ST18 as donor strain for the conjugation procedure, which is a *hemA *mutant of *E. coli *S17 λ-pir [26]. This strain cannot synthesize the general tetrapyrrole precursor aminolevulinic acid (ALA). Hence, to complement the lethal mutation ALA has to be added to the medium for growth. Consequently, for the selection of plasmid-containing *Roseobacter *recipients after conjugation hMB agar plates without ALA were used to inhibit growth of the *E. coli *donor cells. Several conditions of the conjugation procedure were varied including medium composition and conjugation time (for details see Methods section). We found no obvious differences between 24 h- and 48 h-conjugation time (data not shown). Even conjugation times below 24 h might be sufficient for the fast growing *Phaeobacter *strains and *O. indolifex*.

Only two of the tested growth media provided appropriate conditions for donor and recipient strains (see above). Therefore, conjugation was carried out at 30°C on hMB and LB+hs agar plates supplemented with ALA. Media composition revealed a significant effect on conjugation efficiency. ALA supplemented hMB resulted in higher conjugation efficiencies. Various ratios of donor to recipient, related to the optical density of the cultures, were tested (1:1, 2:1, 5:1, 10:1). Best conjugation efficiencies were obtained with ratios of 5:1 and 10:1, ranged between 1 × 10^-6 ^and 2.4 × 10^-2 ^(Table 3). The lowest efficiencies were observed for the *Phaeobacter *and *Roseobacter *strains.

**Table 3:** Conjugation efficiency determined with the vector pBBR1MCS.

Strains	Conjugants/viable cells	Conjugants/ml
*P. inhibens*	1.0 × 10^-6^	1.0 × 10^5^
*P. gallaeciensis*	2.0 × 10^-4^	3.0 × 10^3^
*O. indolifex*	2.7 × 10^-2^	5.0 × 10^5^
*R. litoralis*	5.0 × 10^-4^	1.0 × 10^3^
*R. denitrificans*	2.0 × 10^-4^	2.0 × 10^3^
*D. shibae*	2.4 × 10^-2^	2.0 × 10^6^

^a^The recipient *Roseobacter *strains were cultivated for 18 h in MB at 30°C and the donor *E. coli *ST18 was grown up to the logarithmic phase (OD_578 _= 0.5-0.6) in LB supplemented with 50 μg/ml ALA at 37°C. Mating mixtures were incubated on hMB supplemented with 50 μg/ml ALA over 24 h at 30°C in a donor:recipient ratio 10:1. Afterwards, the cells were resuspended in 1 ml MB, diluted serially in 1.7% (w/v) sea salt solution and plated on hMB with and without antibiotics, respectively, to determine the number of conjugants and viable cells. A donor:recipient ratio of 5:1 revealed the same results. The results represent the mean of three independent experiments performed in duplicate.

Several plasmids were tested for transfer via conjugation. These plasmids were successfully used for homologous expression of genes to complement gene knockouts *in trans *in other Gram-negative bacteria before. The IncP-plasmids pFLP2, pLAFR3 and pUCP20T were not transferable or not stable in the tested *Roseobacter *strains (see below). In contrast, the IncQ-plasmids pRSF1010, pMMB67EH and the tested pBBR1MCS derivates were transferable. They were recovered from exconjugants by plasmid-DNA preparation and subsequently visualized via gel electrophoresis.

### Plasmid Stability

There is only one report about homologous gene expression in *Roseobacter *clade bacteria using the vector pRK415 [21]. This vector was widely used for a broad range of Gram-negative species, including *R. sphaeroides *[e.g. [44,45]]. However, the small numbers of restriction enzyme sites available for cloning and the use of tetracycline as selective marker represent major drawbacks for its use. Therefore, we tested the stability of several mobilizable plasmids of different sizes, carrying different antibiotic resistance genes and origins of replication that belong to distinct incompatibility groups (IncP, IncQ). The maintenance of the plasmids was analysed by spreading cells, which were grown over 10 passages until stationary phase in MB without antibiotics, on hMB agar plates in the presence and absence of antibiotics. Moreover, we tested the cells for the presence of the plasmid by plasmid preparation and visualisation via gel electrophoresis. A reproducible and stable transformation of the *Roseobacter *cells was only obtained with pBBR1MCS derivates. This broad-host-range vector contains the origin of replication of pBBR1 from *Bordetella bronchiseptica*. It has a wide compatibility to IncQ, IncP, IncW, ColE1 and p15A ori plasmids [46,47]. The IncQ containing plasmids pRSF1010 and pMMB67EH were also transferable into the *Roseobacter *bacteria, except for the *Phaeobacter *strains. But in contrast to pRSF1010, pMMB67EH was not stable and got lost after 1 - 2 passages even in the presence of selection pressure. Interestingly, the IncP plasmids pLAFR3, pUCP20T and pFLP2, which are suitable for many other Gram-negative bacteria [48-50], were not transferable or not stable in the tested *Roseobacter *strains.

The members of the *Roseobacter *clade contain up to 13 natural plasmids in a size range of 4.3 - 821.7 kb [4]. For example, *D. shibae *DFL12^T ^type strain contains five plasmids with a size of 72 to 190 kb [51]. Three of the five plasmids harbor a *repABC*-type replicon, one contains a *repA*- and one a *repB*-type replicon [51]. The stability of different plasmids within one cell depends mainly on their incompatibility groups, which are based on the nature of genetic elements involved in plasmid replication or partitioning [15]. Incompatibility is thereby a manifestation of relatedness of these elements, meaning that plasmids with closely related replication origins are incompatible and therefore not stable within one cell [15]. The replicons of the IncP plasmids seem to be closely related to the natural plasmids of the *Roseobacter *bacteria, resulting in the observed instability.

Moreover, at least four of the five plasmids of *D. shibae *contain additional systems for plasmid maintenance. These are composed of two small genes, encoding a stable toxin as well as a less stable antitoxin [51]. The antitoxin must be continually produced to prevent the long-living toxin from killing the cell. Otherwise the toxin induces cell death once the plasmid gets lost during cell division [51,52]. Such toxin/antitoxin systems are characteristic for low copy plasmids and provide plasmid specific differences between various vectors and therefore sustain their compatibility and plasmid replacement protection [53].

### Reporter gene system

**Figure 1:**
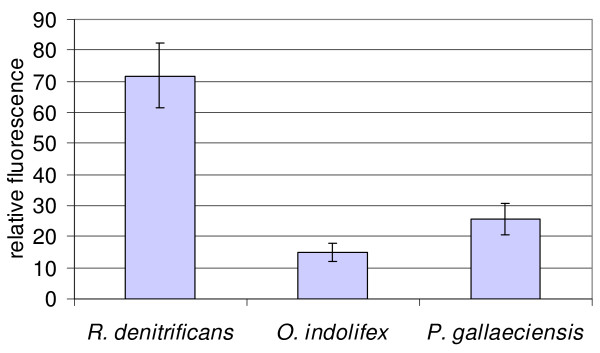
**Flavin-based fluorescent protein as reporter gene**. Fluorescence quantification of pRhokHi-2-FbFP-containing *Roseobacter *bacteria. Liquid cultures (MB, 48 h, 30°C, 200 rpm) were diluted in MB to an OD_578 _of 0.7 and excited at 450 nm in a luminescence spectrometer LS 50 B from Perkin Elmer. The fluorescence emission was detected at 475 - 550 nm. Cultures of wildtype strains were used as a negative control. Results are expressed as mean values of three independent measurements.

Reporter genes are commonly used for the analysis of promoter activities and transcriptional regulation events. A system using *lacZ *reporter gene fusions was recently described for *Sulfitobacter *[23]. Using the novel broad-host-range expression vector pRhokHi-2FbFP [54] based on the conjugally transferable and stably maintained plasmid pBBR1MCS, a novel expression system was established. The vector pRhokHi-2 has been designed for constitutive expression of target genes in the phototrophic bacterium *R. capsulatus*. For the analysis of vector-mediated gene expression a pRhokHi derivative was used harbouring a reporter gene encoding the oxygen-independent, flavin mononucleotide-based fluorescent protein FbFP from *Bacillus subtilis *as reporter [55]. Thus, it is applicable under both aerobic and anaerobic conditions [55]. For many natural habitats oxygen limiting conditions are of central importance. However, the commonly employed reporters including β-galactosidase, luciferase or green fluorescent protein (GFP) require oxygen for dye development, bioluminescence and fluorescence [55]. For a proof of principle the *fbFP *gene was cloned under the control of the constitutive promoter of the aminoglycoside phosphotransferase II (*aphII*) gene in a pBBR1MCS derivate [55]. The plasmid was introduced into the *Roseobacter *strains by conjugation and fluorescence measurement were made of the *fbFP *expressing recipients in comparison to the wildtype strains. Clear emission signals were observed in the range of 15 RFU for *O. indolifex *to 72 RFU for *P. gallaeciensis *at 520 nm upon excitation with blue-light at 450 nm (Figure 1). The altered range of fluorescence might be explained by different copy numbers of the plasmid, different codon usages or different promoter utilisation by the tested strains. The stability and the evenly distribution of pRhokHi-2FbFP within the populations was verified by fluorescence microscopy (data not shown). The observations indicated that FbFP can be used for *in vivo *fluorescence measurements in various *Roseobacter *strains.

### Gene deletion mutants of *D. shibae *DFL12^T^

**Figure 2:**
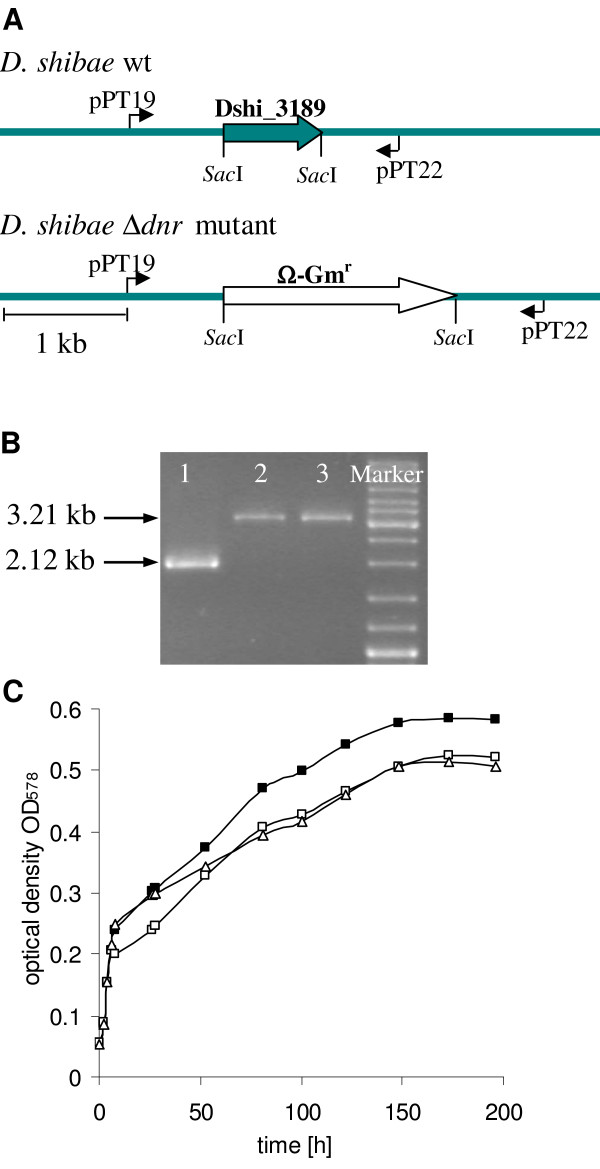
**Gene knockout strategy in *D. shibae *DFL12^T^**. **(A) **Schematic presentation of the *dnr *locus of *D. shibae *DLF12^T ^wildtype and the corresponding Δ*dnr*-mutant. The deletion of Dshi_3189 (*dnr*) after homologous recombination into the *D. shibae *DFL12^T ^genome was confirmed by **(B) **PCR of *D. shibae *DFL12^T ^(line 1) and the Δ*dnr *knockout mutants (line 2 and 3), using the primers oPT19 and oPT22 and by **(C) **growth of *D. shibae *DFL12^T ^and two Δ*dnr *knockout mutants in MB supplemented with 25 mM nitrate under anaerobic conditions at 30°C and 100 rpm. Shown are the growth curves of *D. shibae *DFL12^T ^(-■-), *D. shibae *DFL12Δ*dnr*1 (-□-) and *D. shibae *DFL12Δ*dnr*2 (-Δ-).

The dissimilative nitrate respiration regulator Dnr is a global regulator for anaerobic growth under denitrifying conditions in pseudomonads [56-58]. Six homologous genes were identified in the genome of *D. shibae*. For the construction of a gene deletion mutant of one of these *dnr *genes (Dshi_3189) the vector pEX18Δ*dnr*::Gm^r ^was constructed (Figure 2A). Replacement of the *dnr *gene with the gentamicin cassette was varified via PCR (Figure 2B) resulting in a 2.12 kb fragment for the wildtype corresponding to the 711 bp *dnr *gene, the 652 bp upstream region and the 758 bp downstream region of *dnr*. For the deletion mutants a 3.21 kb fragment was observed containing the up- and downstream regions and the 1.8 kb gentamicin cassette.

Growth behaviour analysis of *D. shibae *DFL12^T ^under anaerobic conditions with nitrate as electron acceptor clearly showed that *D. shibae *was able to grow by denitrification (Figure 2C). This is of special interest, since *D. shibae *was previously described as strict aerobic bacterium [25]. The recently sequenced and annotated genome on *D. shibae *DFL12^T ^recovered clusters of genes necessary for anaerobic metabolism [51].

The comparison of the *D. shibae *wildtype to the obtained *dnr*- mutants revealed a significant reduction of anaerobic nitrate respiratory growth of the tested mutants (Figure 2C), demonstrating the influence of the regulator Dnr on the growth under denitrifying conditions. The presence of six *dnr *genes indicated a fine-tuned regulation of this metabolic pathway. This was confirmed by the minor growth reduction of the *dnr *mutants.

## Conclusion

Genetic tools and methods for transformation and stable plasmid maintenance were established for a variety of *Roseobacter *clade bacteria. A reporter gene system and a chromosomal gene knockout system were based on these methods and applied to selected members of the clade. Since the methods shown here were functional in all of the tested species ranging over the whole phylogenetic tree of the *Roseobacter *clade, an easy and successful transfer to other members of this group can be proposed.

Initial experiments with a *dnr *mutant of *D. shibae *showed an influence of this regulator on the growth under denitrifying conditions.

## Methods

### Bacterial strains, plasmids and growth conditions

Strains used in this study are described in Table 4. Table 5 shows the used plasmids. The *Escherichia coli *strain ST18 was cultured in Luria-Bertani (LB) medium prepared of 10 g tryptone, 5 g yeast extract and 10 g NaCl in 1 L H_2_O dest., supplemented with 50 μg/ml aminolevulinic acid (ALA, Sigma-Aldrich, Munich, Germany) at 37°C and 200 rpm as described before [26]. The marine bacteria of the *Roseobacter *clade were usually cultured in the commercial available Marine Broth (MB, Roth) at 30°C and 200 rpm. For the preparation of half-concentrated MB (hMB) 20.05 g media were dissolved in 1 l H_2_O dest.. After autoclaving, MB containing media were sterile filtered to remove precipitates. LB containing 17 g sea salts (Sigma-Aldrich, Munich, Germany; LB+s) and LB supplemented with 8.5 g sea salts (LB+hs) were prepared for the determination of the optimal growth conditions of the *Roseobacter *bacteria. For the preparation of agar plates 1.5% (w/v) agar (Roth, Karlsruhe, Germany) were added and dissolved by heating prior to autoclaving. For anaerobic growth, MB was supplemented with 25 mM nitrate. Anaerobic flasks were used for incubation at 30°C and 100 rpm.

**Table 4:** Bacterial strains used in this study.

Strains	Origin/description	Reference
*Escherichia coli *ST18	S17-1Δ*hemA thi pro hsdR*^-^*M*^- ^with chromosomal integrated [RP4-2 Tc::Mu:Km^r^::Tn7, Tra^+ ^Tri^r ^Str^r^]	[26]
*Escherichia coli *DH5α	*endA1 hsdR1*[r_K_^- ^m_K_^+^] *glnV44 thi-1 recA1 gyrA relA *Δ[*lacZYA-argF*)*U169 deoR *[Φ80*dlac *Δ[*lacZ*]*M15*)	[62]
*Phaeobacter inhibens *T5^T^	type strain DSM16374^T^	[24]
*Phaeobacter gallaeciensis *2.10	wild type	[24,63]
*Oceanibulbus indolifex *HEL-45^T^	isolated from a sea water sample, type strain, DSM14862^T^	[64]
*Roseobacter litoralis *6996^T^	type strain, DSM6996^T^	[9]
*Roseobacter denitrificans *7001^T^	type strain, DSM7001^T^	[9]
*Dinoroseobacter shibae *DFL-12^T^	isolated from the dinoflagellate *Prorocentrum lima*, type strain, DSM16493^T^	[25,51,65]
*Dinoroseobacter shibae *DFL-16	isolated from the dinoflagellate *Alexandrium ostenfeldii*	[65]
*Dinoroseobacter shibae *DFL-27	isolated from the dinoflagellate *Alexandrium ostenfeldii*	[25,65]
*Dinoroseobacter shibae *DFL-30	isolated from the dinoflagellate *Alexandrium ostenfeldii*	[65]
*Dinoroseobacter shibae *DFL-31	isolated from the dinoflagellate *Alexandrium ostenfeldii*	[65]
*Dinoroseobacter shibae *DFL-36	isolated from the dinoflagellate *Alexandrium ostenfeldii*	[65]
*Dinoroseobacter shibae *DFL-38	isolated from the dinoflagellate *Alexandrium ostenfeldii*	[65]

^T ^DSMZ type strain

**Table 5:** Plasmids used in this study.

Plasmids	Description	Reference
pFLP2	9.4 kb IncP Amp^r ^Flp recombinase ori1600 oriT	[48]
pLAFR3	22.0 kb IncP Tet^r ^RP4	[50]
pUCP20T	4.17 kb IncP Amp^r ^P_lac _ori1600 oriT	[49]
pRSF1010	8.7 kb IncQ Sm^r ^Su^r ^*repA repB repC*	[66]
pMMB67EH	8.8 kb IncQ Amp^r ^*lacI*^*q *^P_tac _rrnB oriV oriT	[67]
pBBR1MCS1^ab^	4.72 kb Cm^r ^*lacZ *P_lac _P_T7 _*rep*	[46]
pBBR1MCS2^ab^	5.14 kb Km^r ^*lacZ *P_lac _P_T7 _*rep*	[47]
pBBR1MCS3^ab^	5.23 kb Tet^r ^*lacZ *P_lac _P_T7 _*rep*	[47]
pBBR1MCS4^ab^	4.95 kb Amp^r ^*lacZ *P_lac _P_T7 _*rep*	[47]
pBBR1MCS5^ab^	4.77 kb Gm^r ^*lacZ *P_lac _P_T7 _*rep*	[47]
pRhokHi-2-FbFP	7.38 kb Cm Km P_T7 _*FbFP *under control of P_aphII _constructed from pBBR1MCS1	[54,55]
pEX18Ap	5.8 kb Ap^R^, *oriT*^+^, *sacB*^+^, *lacZα*, suicide vector	[48]
pPS858	4.5 kb Ap^R^, Gm^R^, GFP^+^	[48]

^a^The derivates of the pBBR1MCS plasmid are compatible with IncQ, IncP, IncW, ColE1 and p15A ori.

^b^Different derivates of pBBR1MCS were used in the different *Roseobacter *strains in dependence on their antibiotic susceptibilities.

### Determination of the minimal inhibitory concentration

For the determination of minimal inhibitory concentrations (MIC) 5 ml hMB was supplemented with freshly prepared antibiotic solutions from 0 - 500 μg/ml in 5 μg steps. Test tubes were inoculated with cultures of the respective bacterial strains grown for 24 h in MB to an OD_578 _of 0.05. Subsequently, bacterial growth was checked by OD_578 _measurements after incubation for 3 and 5 days at 30°C without shaking. The MIC is defined as the lowest concentration of a tested antibiotic, which inhibits the growth of bacteria. All experiments were repeated three times in duplicate. The used antibiotics were obtained from manufactures as followed: ampicillin (Roth, Karlsruhe, Germany), carbenicillin disodium salt (Gerbu Biotechnik GmbH, Gaiberg, Germany), chloramphenicol (Roth, Karlsruhe, Germany), gentamicin sulphate (Roth, Karlsruhe, Germany), kanamycin sulfate (Gerbu Biotechnik GmbH, Gaiberg, Germany), spectinomycin dichloride pentahydrate (Sigma-Aldrich, Munich, Germany), streptomycin sulphate (United States Biochemical Corp., Cleveland, USA), tetracycline hydrochloride (United States Biochemical Corp., Cleveland, USA). For selection of plasmid-containing *Roseobacter *recipients on agar plates after conjugation the twofold concentration of the MIC of the respective antibiotic in hMB was used.

### Preparation of chemically competent cells for the transfer of plasmid-DNA into *Roseobacter *strains

Chemo-competent cells were prepared as described by Sambrook et al. [1989]. To prepare CaCl_2_- competent cells, the *Roseobacter *strains were cultivated in MB at 30°C and 200 rpm up to an OD_578 _of 0.7. Ten ml of the culture were centrifuged for 15 min at 3,200 × g and 4°C. The bacterial pellet was resuspended in 2 ml cold 10% (v/v) glycerol with 100 mM CaCl_2 _in ultra-pure water and centrifuged for 2 min at 8,000 × g and 4°C. Afterwards, the cells were resuspended in 100 μl cold 10% (v/v) glycerol with 100 mM CaCl_2 _in ultra-pure water and incubated on ice for 1 h. Subsequently, 200 μl aliquots were frozen in liquid nitrogen and stored at -80°C.

To prepare RbCl_2_-competent cells, the *Roseobacter *strains were cultivated in 20 ml MB supplemented with 400 μl of a stock solution containing 500 mM MgCl_2 _and 500 mM MgSO_4 _at 30°C and 200 rpm up to an OD_578 _of 0.7. Four ml of the culture were centrifuged for 2 min at 8,000 × g and 4°C. Cells were resuspended in 2 ml ice cold transformation buffer (100 mM CaCl_2_, 50 mM RbCl_2_, 40 mM MnCl_2_) and incubated on ice for 30 min, followed by a centrifugation step for 2 min at 8,000 × g and 4°C. Finally, cells were resuspended in 200 μl transformation buffer. The chemo-competent cells were stored on ice until they were used or frozen at -80°C in 20% (v/v) glycerol.

For the transformation, 200 μl of chemo-competent cells (CaCl_2_- or RbCl_2_-competent) were gently mixed with 50 ng plasmid-DNA and incubated for 30 min on ice. After a heat shock for 2 min at 42°C, 800 μl MB medium was added and the bacteria were incubated for 3 h at 30°C for the expression of the antibiotic resistance marker encoded by the plasmid. Afterwards the cells were sedimented by centrifugation for 2 min at 8,000 × g and 4°C and the supernatant was decanted. Cells were resuspended in the residual liquid and spread on hMB agar supplemented with the appropriate antibiotic.

### Transfer of plasmid-DNA into *Roseobacter *strains by electroporation

Electro-competent cells were prepared as described previously by Miller and Belas [2006] with slight modifications. Therefore, cells were grown in MB medium at 30°C and 200 rpm to an OD_578 _of 0.5. Ten ml culture was centrifuged for 15 min at 3,200 × g. Sedimented cells were washed 5 times with 10 ml cold 10% (v/v) glycerol in ultra-pure water. Then, the cell pellet was resuspended in 400 μl 10% (v/v) glycerol and 50 μl aliquots were frozen in liquid nitrogen and stored at -80°C. For electroporation, 25 - 50 ng plasmid-DNA were added to 50 μl competent cells in an ice cold 2 mm pulser cuvette (Bio-Rad, Munich, Germany). The mixture was treated in a Bio Rad gene pulser II with field strength of 1.5 - 3.0 kV, resistance of 200 Ω and capacitance of 25 μF. After electroporation the cells were transferred to 1 ml cold MB media and incubated overnight at room temperature with shaking at 300 rpm to allow the expression of antibiotic resistance genes. To investigate electroporation efficiency, cells were serially diluted in 1.7% (w/v) sea salt solution and plated on hMB agar plates with the appropriate antibiotic concentration and incubated for 2 days (*Phaeobacter *strains and *O. indolifex*) or 4 days (*Roseobacter *strains and *D. shibae*) at 30°C. Subsequently, colony forming units (cfu) were determined.

### Conjugal transfer of plasmid-DNA from *E. coli *into *Roseobacter *strains

The conjugation procedure was modificated for *Roseobacter *strains from the protocol of Thoma and Schobert [2009]. The recipient *Roseobacter *strains were cultivated for 18 h in MB-Medium. The donor *E. coli *strain ST18 was grown in LB-medium supplemented with 50 μg/ml ALA (Sigma-Aldrich, Munich, Germany) up to the logarithmic phase (OD_578 _= 0.5 - 0.6). Both cultures were mixed in a donor:recipient ratio of 1:1; 2:1; 5:1 or 10:1 according to the optical density (OD_578_) of the cultures. Cells were sedimented by centrifugation for 2 min at 8,000 × g at 20°C, resuspended in the residual liquid and used to inoculate hMB agar, LB+hs agar and hLB+hs agar respectively, all supplemented with 50 mg/ml ALA, in form of a spot. The plates were incubated at 30°C for 24 h and 48 h. Subsequently, cells were scraped from the plate and resuspended in 1 ml MB by vigorous shaking. Disruption of cell aggregates was confirmed via microscopic inspection of the resulting single cells. A dilution series in 1.7% (w/v) sea salt solution was prepared and plated on hMB with the appropriate antibiotic concentration to determine the number of transconjugants per ml. Since the plates did not contain ALA the auxotrophic donor *E. coli *strain was not able to grow. In parallel, transconjugants were also plated on hMB without antibiotics to determine the number of viable cells per ml. The efficiency of conjugation was defined as the ratio between conjugants:viable cells. Moreover, a minimum of 10 exconjugants were tested for the presence of the plasmid by plasmid-DNA isolation and gel electrophoresis.

### Isolation of plasmid-DNA from the *Roseobacter *strains

Plasmids used in this study were isolated using the mini plasmid isolation kit from Qiagen (Qiagen, Hilden, Germany) following the manufacturers' instructions for Gram-negative bacteria.

### Genome analysis and bioinformatics approach

For genome analysis of the *Roseobacter *strains the databases of the Joint Genome Institute http://www.jgi.doe.gov [35] and the *Roseobacter *database http://rosy.tu-bs.de/ [12] were used. Open reading frames were identified using BLASTX analysis with a cutoff E value of 1e^-5^. β-lactamase and aminoglycoside resistance genes from *P. aeruginosa *and *E. coli *were used for the study. Moreover, Pfam [59], TIGRfam [60] and COG [61] predictions were used to identify functional homologues.

The genome of *D. shibae *DFL12^T ^was sequenced at the Joint Genome Institute (JGI) Production Genomics Facility. The sequences, comprising a chromosome and 5 plasmids, can be accessed using the GenBank accession numbers NC_009952, NC_009955, NC_009956, NC_009957, NC_009958 and NC_009959. Manual curation and reannotation of the genome was carried out using the Integrated Microbial Genomes Expert Review System (img/er http://imgweb.jgi-psf.org) [51] and the Artemis software package http://www.sanger.ac.uk/Software/Artemis/v9.

The complete and annotated genome sequences of *Roseobacter denitrificans *strain OCh 114 and its associated plasmids have been deposited in the DDBJ/EMBL/GenBank database under accession numbers CP000362, CP000464, CP000465, CP000466, and CP000467. Initial annotation data were built using the Annotation Engine at The Institute for Genomic Research http://www.tigr.org/edutraining/training/annotation_engine.shtml, followed by comprehensive manual inspection and editing of each feature by using Manatee http://manatee.sourceforge.net/.

### Fluorescence imaging of reporter gene carrying cells

Some of the *Roseobacter *clade members were fluorescence labelled using the FMN-based fluorescence protein FbFP [55] (available as evoglow-Bs1 from Evocatal GmbH, Düsseldorf, Germany). Therefore, the fluorescence reporter gene was constitutively expressed using the broad-host-range expression vector pRhokHi-2-FbFP [54]. Fluorescence microscopy was used for *in vivo *fluorescence imaging of living cells. An aliquot of the microbial cell culture was placed on a microscope slide and illuminated with light of the wavelength 455-495 nm. Fluorescence emission of single cells was analyzed in the ranges of 500-550 nm using a Zeiss Fluorescence Microscope (Axiovert 200 M) at a magnification of 600-fold. Documentation was carried out using the camera AxioCam (Carl Zeiss, Jena, Germany) and the software AxioVision Rel 4.5 (Carl Zeiss, Jena, Germany).

### Quantification of reporter gene activity

Liquid cultures (MB, 48 h, 30°C, 200 rpm) of the pRhokHi-2-FbFP containing *Roseobacter *strains were diluted in MB to an OD_578 _of 0.7 and excited with light of the wavelength of 450 nm. Fluorescence emission was detected at 475 - 550 nm. Cultures of the wildtype strains served as negative control. For quantification of the fluorescence the luminescence spectrometer LS 50 B (Perkin Elmer, Waltham, Massachusetts, USA) was used.

### Construction of *D. shibae *DFL12^T ^dnr (Dshi_3189) deletion mutants

To obtain gene deletion mutants from *D. shibae *DFL12^T^, the well-established suicide vector for Gram-negative bacteria pEX18Ap was used [48]. To construct the gene deletion vector pEX18Δ*dnr*::Gm^r^, the *Sac*I-digested Ω-gentamicin resistance cassette of pPS858 [48] was cloned between two PCR fragments of the *dnr *gene (Dshi_3189) in the multiple cloning site of pEX18Ap. The two PCR fragments contained DNA homologous to upstream and downstream regions of the Dshi_3189 gene. A 652-bp fragment containing the upstream promoter region of Dshi_3189 was amplified using primer oPT19 (5'-GGGGTACCAATGCCATGACCT ACTTC-3'), which contains a *Kpn*I restriction site at the 5' end, and oPT20 (5'-CGAGCTCCGCATGAACGAGTCATCTT-3'), containing a *Sac*I site (both restriction sites underlined). The primers oPT21 (5'-CGAGCTCAGCAGAACCATGCGGAGAT-3'), containing a *Sac*I site, and oPT22 (5'-CCCAAGCTTTCACCAGCGGGCTTTTC-3'), which contains a *Hind*III site (both restriction sites underlined), amplified 758 bp of the corresponding downstream region of Dshi_3198. The suicide vector pEX18Δ*dnr*::Gm^r ^was used to replace the Dshi_3198 gene with the Ω-gentamicin cassette. To confirm homologous recombination PCR analysis was performed. Furthermore, the growth behaviour of the resulting mutants was analysed under anaerobic conditions with nitrate as electron acceptor, as outlined before [57].

## Abbreviations

ALA: aminolevulinic acid; Amp: ampicillin; Cm: chloramphenicol; FbFP: flavin mononucleotide-based fluorescent protein; Gm: gentamicin; hMB: half concentrated Marine Broth; Inc: incompatibility group; Km: kanamycin; LB+hs: LB supplemented with 8.5 g sea salts; LB+s: LB supplemented with 17 g sea salts; MB: Marine Broth; OD: optical density; RFU: relative fluorescence unit; Sm: streptomycin; Spec: spectinomycin; Su: sulfonamide; Tet: tetracycline

## Authors' contributions

DJ and PT conceived the study. PT supervised the work. IB made initial experiments of the antibiotic resistance screening and the conjugation approaches. TP optimized the conjugation method and was responsible for further antibiotic resistance screenings, the establishment of the gene knockout strategy and the reporter gene fusion system. PT developed the electroporation method and the chemical transformation. TP and PT drafted the manuscript. DJ edited the manuscript. IWD, TD and MS provided strains, plasmids and helpful discussions on ecological and genetic questions. All authors read, commented on and approved the final manuscript.
